# A Flow Cytometry Method for Rapidly Assessing Mycobacterium tuberculosis Responses to Antibiotics with Different Modes of Action

**DOI:** 10.1128/AAC.02712-15

**Published:** 2016-06-20

**Authors:** Charlotte Louise Hendon-Dunn, Kathryn Sarah Doris, Stephen Richard Thomas, Jonathan Charles Allnutt, Alice Ann Neville Marriott, Kim Alexandra Hatch, Robert James Watson, Graham Bottley, Philip David Marsh, Stephen Charles Taylor, Joanna Bacon

**Affiliations:** a Public Health England, National Infection Service, Porton Down, Salisbury, Wiltshire, United Kingdom; b National Institute for Biological Standards and Control, South Mimms, Potters Bar, Hertfordshire, United Kingdom; c InCytometry, Todenham, Gloucestershire, United Kingdom

## Abstract

Current methods for assessing the drug susceptibility of Mycobacterium tuberculosis are lengthy and do not capture information about viable organisms that are not immediately culturable under standard laboratory conditions as a result of antibiotic exposure. We have developed a rapid dual-fluorescence flow cytometry method using markers for cell viability and death. We show that the fluorescent marker calcein violet with an acetoxy-methyl ester group (CV-AM) can differentiate between populations of M. tuberculosis growing at different rates, while Sytox green (SG) can differentiate between live and dead mycobacteria. M. tuberculosis was exposed to isoniazid or rifampin at different concentrations over time and either dual stained with CV-AM and SG and analyzed by flow cytometry or plated to determine the viability of the cells. Although similar trends in the loss of viability were observed when the results of flow cytometry and the plate counting methods were compared, there was a lack of correlation between these two approaches, as the flow cytometry analysis potentially captured information about cell populations that were unable to grow under standard conditions. The flow cytometry approach had an additional advantage in that it could provide insights into the mode of action of the drug: antibiotics targeting the cell wall gave a flow cytometry profile distinct from those inhibiting intracellular processes. This rapid drug susceptibility testing method could identify more effective antimycobacterials, provide information about their potential mode of action, and accelerate their progress to the clinic.

## INTRODUCTION

Tuberculosis (TB) is one of the most serious bacterial infections; in 2013, the World Health Organization estimated that there were 9 million new TB cases and 1.5 million TB deaths worldwide (1). There is an urgent need for more effective interventions for the disease, including more effective vaccines, better diagnostics, and improved antibiotics. Currently, treatment of TB requires a combination of antimycobacterial drugs for at least 6 months. It is important that efforts be focused on developing new compounds that are effective at sterilizing multidrug-resistant (MDR) TB, latent TB, and phenotypic drug-tolerant Mycobacterium tuberculosis to reduce the drug treatment time and to minimize relapse of disease (2). *In vitro* drug susceptibility tests that can rapidly determine the activity of antimicrobials against M. tuberculosis are required for the optimization of new antibiotic combinations and at the same time identify M. tuberculosis subpopulations that are challenging to culture (3).

Current methods, such as the conventional proportion method (4) and even the new rapid liquid culture techniques (5), do not capture information about organisms that are not immediately culturable after drug exposure (6). These traditional methods, therefore, can provide a misleading readout of drug inhibition and cell death, and it can take several weeks of incubation between the time of drug exposure and the confirmation of sensitivity or resistance.

There has been much debate about the existence of viable but nonculturable (VBNC) bacteria and their phenotypic characteristics. VBNC cells have been characterized by the loss of culturability on agar, which can lead to the inaccurate enumeration of cells within a bacterial population. It has previously been highlighted that the capacity of a cell to replicate and produce viable colonies on agar is directly reflective of its viability and that the VBNC state is a self-contradictory expression (7). However, this simply appears to be a question of terminology and a failure to provide a working definition in each case. There are situations where bacteria can achieve a state of nonculturability postexposure to environmental stresses, such as starvation or oxygen limitation, requiring specific conditions for resuscitation or regrowth (8–10).

The aim of our study was to develop a more rapid flow cytometry tool for determining antibiotic activity against M. tuberculosis within a few hours of drug exposure using fluorescent dyes and to capture information about the susceptibility of the whole population and not just those bacteria that can grow in media postexposure. The fluorescent dyes used were calcein violet (CV) with an acetoxy-methyl ester (AM) group (CV-AM) and Sytox green (SG). CV-AM is used for distinguishing live cells through the action of intracellular esterase activity (11, 12). CV can easily be excited with a violet laser, allowing other laser lines to be used for conventional fluorochromes. We also selected SG, which permeates damaged bacteria and binds to DNA (13). It does not cross intact membranes but easily penetrates compromised membranes, which are characteristic of dead cells. It exhibits more than a 500-fold fluorescence enhancement upon binding to nucleic acids. Alternative dyes and flow cytometry methods have been assessed for their utility in measuring the viability of mycobacterial species and for testing antibiotic activity against M. tuberculosis but have not been routinely adopted (14). Previously, antibiotic susceptibility testing has been demonstrated with the use of other metabolic dyes, such as 5-chloromethylfluorescein diacetate (FDA) (15). Syto 16 is a nucleic acid stain that more readily penetrates dead cells than other dyes and was formerly used to study the growth cycle of Mycobacterium avium (16). Pina-Vaz et al. (17) used Syto 16 for studying the susceptibility of M. tuberculosis to multiple antibiotics, including rifampin and isoniazid, using flow cytometry.

Other phenotypic techniques that have recently been introduced have been shown to detect growth more rapidly than the conventional proportion method (4) by measuring the metabolic activity of dividing bacteria. Rapid liquid culture-based techniques that can detect growth-dependent changes, such as CO_2_ production (the Bactec 460 and MB/BacT systems) (18), or that can fluorometrically detect oxygen consumption, such as the mycobacterial growth indicator tube (MGIT 960 system) (19), have been established and used in clinical laboratories. However, the incubation time can still be more than 14 days. Other assays measure the ability of living bacteria to convert an oxidation-reduction color indicator dye, resazurin, to the reduced state or the ability of M. tuberculosis to reduce nitrate to nitrite in the nitrate reductase assay (20, 21). Reporter strains enable assessment of the metabolic state, bacterial heterogeneity, and bactericidal or bacteriostatic activity of drugs (22, 23). This reporter technology has also been combined with fluoromycobacteriophages to deliver green fluorescent protein marker genes into M. tuberculosis, which can then be monitored by flow cytometry or fluorescence microscopy (24, 25). The flow cytometry method (flow method) that we present here is an alternative approach for dissecting antibiotic-treated populations and characterizing subpopulations that may not be detected by growth in medium postantibiotic exposure. M. tuberculosis cell samples from fast-growth (mean generation time [MGT], 23.1 h) chemostats were exposed to either rifampin or isoniazid over a range of concentrations, and flow cytometry analyses and viable counts were used to determine the responses to these antibiotics. The results demonstrated that the sterilizing activity of isoniazid was concentration dependent, whereas that of rifampin was time dependent. We made qualitative observations that intracellularly targeted profiles and cell wall-targeted profiles could be differentiated by the pattern of responses in their fluorescence profiles. These distinctive patterns of fluorescence pointed to the use of the flow method for determining the mode of action of novel antibiotics, particularly those that target the cell wall, and indicated that this information could accelerate their progress to the clinic for the treatment of either MDR or drug-tolerant persistent subpopulations.

## MATERIALS AND METHODS

### Reagents.

Primary stock solutions of rifampin, isoniazid, ciprofloxacin, kanamycin, and BTZ-043 were made at 10 mg ml^−1^ in 100% dimethyl sulfoxide (DMSO). These were frozen in aliquots (100 μl) at −20°C. A working stock of rifampin at 1 mg ml^−1^ (diluted from the 10-mg ml^−1^ stock with water) was also frozen at −20°C. At the point of use, the stocks were diluted to the desired concentration with water and then filter sterilized (pore size, 0.2 μm).

The dyes CV-AM and SG were prepared in the following way. Each aliquot of CV-AM received (catalogue no. C34858; Invitrogen, Life Technologies) was dissolved in 25 μl of DMSO. Freshly dissolved CV-AM was used on each occasion. SG (catalogue no. S7020; Invitrogen, Life Technologies) was diluted from the manufacturer's stock solution of 5 mM to a working solution of 20 μM in DMSO. Fresh aliquots (stored in opaque vials at −20°C) were used on each occasion.

### Strains and medium.

Studies were performed with M. tuberculosis strain H37Rv (NCTC catalog no. 7416). Stock cultures were grown on Middlebrook 7H10 with oleic acid-albumin-dextrose-catalase (OADC) for 3 weeks at 37°C ± 2°C.

### Chemostat growth of M. tuberculosis.

M. tuberculosis was grown in continuous culture using a chemostat and Centre for Applied Microbiology Research mycobacterium medium Mod2 (CMM Mod2) (26). Cultures were performed in a 1-liter fermentation vessel with a working volume of 500 ml. The culture system was operated as a chemostat by controlling addition of the growth-limiting nutrient, glycerol, at a constant dilution rate of either 0.03 h^−1^ or 0.01 h^−1^ to give an MGT of 23.1 h or 69.3 h, respectively (27, 28). The culture was agitated by a magnetic bar placed in the culture vessel coupled to a magnetic stirrer positioned beneath the vessel. Culture conditions were continuously monitored by an Anglicon Microlab fermentation system (Brighton Systems) linked to sensor probes inserted into the culture through sealed ports in the top plate. The vessel was filled with 500 ml of sterile culture medium (CMM Mod2), and the parameters were allowed to stabilize at 37°C ± 1°C; pH 6.9 ± 0.3 (the pH was monitored using a gel-filled pH electrode from Mettler-Toledo); and a dissolved oxygen tension (DOT) of approximately 50% air saturation (10% DOT), which was monitored by a polarographic oxygen electrode (Broadley James, United Kingdom). A dense inoculum was prepared by resuspending colonies from 5 Middlebrook agar cultures (grown at 37°C ± 1°C for 3 weeks) in sterile deionized water. The inoculum was aseptically transferred to the culture vessel, to provide an initial culture turbidity of approximately 0.25 at 540 nm. The culture temperature was monitored by a Pt100 temperature probe (Anglicon) and maintained at 37°C ± 1°C by use of a heating pad positioned beneath the culture vessel. Cultures were allowed to continue for a mean of 5 generations after the turbidity stabilized before commencing steady-state sample collection. Cell samples were regularly removed to monitor growth and survival and for the preparation of heat-killed cells, cell staining, and antibiotic exposure experiments.

### Growth and survival.

Bacterial growth and survival were assessed by determining the number of viable cells in each culture system at specific time points. Each sample was decimal diluted in sterile water, and 100-μl aliquots were plated onto Middlebrook 7H10 plates with OADC in triplicate. The plates were incubated at 37°C for up to 4 weeks before enumeration of the colonies that formed. The plating method of Miles et al. (29) was used to determine the viability of bacteria exposed to antibiotic. Serial decimal dilutions down to 10^−6^ were performed in phosphate-buffered saline (PBS). Three spots each consisting of 20 μl of diluted cells were dispensed onto each quarter of a divided plate. The plates were left undisturbed for 1 to 2 h to allow the spots to dry. The colonies on the plates were counted after 2 weeks of incubation at 37°C. The lower limit of detection for these plates was 50 CFU ml^−1^, which corresponds to an average of 1 colony in each of the three spots.

### Staining of cells at different growth rates sampled from chemostats.

One milliliter of cells at either growth rate was sampled from the chemostat and adjusted to a turbidity of 0.5 (at 540 nm) using growth medium. Five microliters of the CV-AM stock solution was then added to 1 ml of the cells, and the cells were incubated at 37°C for 1 h while shaking at 220 rpm. Following this, the cell samples were spun at 6,000 rpm for 5 min, the supernatant was removed, and the resulting pellet was resuspended in 1 ml of Hanks' balanced salt solution (HBSS) buffer containing 4% formaldehyde to kill viable mycobacteria. The cells were left overnight to fix them prior to flow cytometry.

### Antibiotic susceptibility time course. (i) Four-day antibiotic exposure.

Bacteria grown in a chemostat at a dilution rate of 0.03 h^−1^ (MGT, 23.1 h) were sampled, and the optical density (OD) was measured at a wavelength of 540 nm (OD_540_). The bacteria were diluted to an OD_540_ of 0.05 in prewarmed (37°C) CMM Mod2. Five milliliters of the diluted culture was used to inoculate universal tubes containing isoniazid or rifampin to achieve final concentrations of 0 μg ml^−1^, 0.25 μg ml^−1^, 0.5 μg ml^−1^, 1 μg ml^−1^, 2 μg ml^−1^, 4 μg ml^−1^, 8 μg ml^−1^, 16 μg ml^−1^, and 32 μg ml^−1^ for isoniazid and 0 μg ml^−1^, 0.004 μg ml^−1^, 0.008 μg ml^−1^, 0.016 μg ml^−1^, 0.032 μg ml^−1^, 0.128 μg ml^−1^, 0.5 μg ml^−1^, 2 μg ml^−1^, 8 μg ml^−1^, 16 μg ml^−1^, and 32 μg ml^−1^ for rifampin. The universal tubes were incubated at 37°C for 4 days with shaking at 200 rpm. Every 24 h, 450 μl of culture was taken for CV-AM and SG staining and to measure drug susceptibility using the method of Miles et al. (29).

### (ii) Time-dependent assessment of antibiotic activity.

The preliminary 4-day exposure was repeated with fewer antibiotic concentrations for isoniazid (0 μg ml^−1^, 0.25 μg ml^−1^, 0.5 μg ml^−1^, 2 μg ml^−1^, 8 μg ml^−1^, 32 μg ml^−1^) and rifampin (0 μg ml^−1^, 0.016 μg ml^−1^, 0.032 μg ml^−1^, 0.128 μg ml^−1^, 0.5 μg ml^−1^, 32 μg ml^−1^) that spanned the range of concentrations used in the preliminary experiments and included sub-MICs, the MIC, and high concentrations. After 4 days of antibiotic exposure, the cells were spun at 3,000 × *g* and the supernatant was removed. The bacterial pellet was washed twice in PBS, resuspended in an equivalent volume of fresh medium, and incubated for a further 7 days at 37°C with shaking at 200 rpm. Drug exposure was also performed for an extended 11-day period. At 0, 2, 4, 7, and 11 days throughout the 11-day time course, 450 μl of culture was sampled for CV-AM and SG staining and to measure drug susceptibility using the method of Miles et al. (29).

### Susceptibility to kanamycin, ciprofloxacin, and BTZ-043.

Drug exposure was performed over an 11-day period with 3 concentrations of kanamycin (0.5 μg ml^−1^, 2.0 μg ml^−1^, 8.0 μg ml^−1^), ciprofloxacin (0.125 μg ml^−1^, 0.5 μg ml^−1^, 2.0 μg ml^−1^), and BTZ-043 (0.7 ng ml^−1^, 1.4 ng ml^−1^, 6.0 ng ml^−1^). These concentrations are equivalent to 0.25× MIC, 1× MIC, and 4× MIC, respectively, determined from published MIC levels (30–33). Samples were taken at 0, 2, 4, 7, and 11 days and analyzed as described above for isoniazid and rifampin.

### Staining of antibiotic-exposed cells.

One hundred microliters of bacteria in each well of a 96-well microtiter plate was stained with 0.5 μl CV-AM and 1 μl SG (20 μM), and the plate was incubated at 37°C for 1 h in the dark. An unstained sample was treated similarly to provide a control. Further controls to aid with the subsequent gating strategy (Table 1; see Fig. 2) included heat-killed bacteria that were either unstained, stained with SG, or dual stained with CV-AM and SG. These controls enabled the placement of gate P4 (Table 1; see Fig. 2) and verification that there was no spillover of fluorescence from SG staining into the CV channel (450/50 band pass filter [BP]), which was used to measure CV-AM fluorescence intensity. Controls containing zero levels of antibiotic were stained with CV-AM only, as well as dual stained; this enabled the placement of population gate P2 (Table 1; see Fig. 2) and verified that there was no spillover of fluorescence from CV-AM staining into the SG channel (530/40 BP), which was used to measure SG fluorescence intensity. After staining, the bacteria were spun by centrifugation at 2,885 × *g* for 2 min and resuspended in HBSS containing 4% (vol/vol) formaldehyde. A fixation time of 30 min was found to be sufficient for the sterilization of M. tuberculosis (at an OD_540_ of 0.5) to allow removal from biosafety containment level 3 for flow cytometry analyses.

**TABLE 1:** Description of each population gate and the controls used for the gating strategy^*a*^

Gate	Description	Controls used for gating strategy
P1	Unstained cells	All unstained samples; the gate was positioned on both axes to ensure >95% cells in the gate
P2	Calcein positive, SG negative (live)	Dual-stained and no-drug controls stained with CV-AM only; the gate was positioned on the SG axis to include the entire discrete live cell population and on the CV axis to exclude unstained cells (see gate P1)
P3	Calcein positive, SG positive	Remaining space in the top right population gate of the dot plot
P4	SG positive, calcein negative (dead)	Heat-killed cells consisting of both cells stained with SG only and cells dual stained with SG and CV-AM; the gate was positioned on the CV axis to include the entire discrete dead cell population and on the SG axis to exclude unstained cells (see gate P1)

a An image of a dual-parameter dot plot with associated gates P1 to P4 can be viewed in Fig. 2.

### Flow cytometry and analysis.

Bacteria were examined using a CyAn ADP (9-color) analyzer (Beckman Coulter) with an attached Cytek plate loader. Two lasers with excitatory wavelengths of 488 nm and 405 nm were used. SG fluorescence emission (excitation and emission, 488 nm and 523 nm, respectively) was detected in the SG channel (530/40 BP), and CV-AM fluorescence (excitation and emission, 400 nm and 452 nm, respectively) was detected in the CV channel (450/50 BP). Unstained control samples were first run through the flow cytometer to set a population gate around the bacteria to be analyzed by using the forward scatter-versus-side scatter parameters. The voltages in the SG and CV channels were then adjusted so that the fluorescence histogram of the unstained bacteria appeared within the first order of the logarithmic scale of fluorescence. The remaining stained samples were then analyzed on these settings. Ten thousand events were collected at a set standard low event rate. Summit software (version 4.3) was used to analyze the acquired data to create one-parameter fluorescence histogram overlays and two-parameter dot plots. For all fluorescence dot plots, the populations were gated around the unstained control in relation to CV-AM fluorescence and gated around the stained live/zero drug control in relation to SG fluorescence (Table 1). Polygonal population gates were placed according to the position of the populations of cells from the various controls. The percentages of the total cell population residing in each gate were obtained (using Summit software, version 4.3) for each antibiotic concentration and time point. A description of each population gate can be found in Table 1 (see also Fig. 2A). An average for each value from 3 experiments was taken and plotted. Statistical analyses were performed using SigmaPlot software (version 13; Systat Software, San Jose, CA).

## RESULTS

### Calcein violet-AM stains viable M. tuberculosis cells.

**FIG 1:**
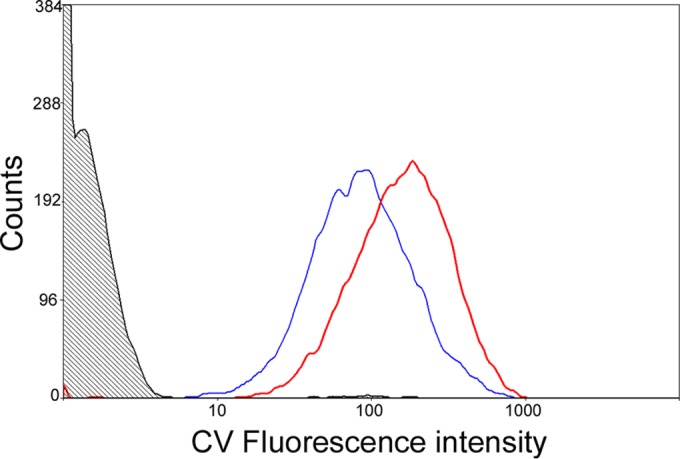
Representative overlay plots showing CV-AM staining of Mycobacterium tuberculosis grown at either a high growth rate (doubling time, 23 h; red line) or a low growth rate (doubling time, 69 h; blue line). The shaded area represents the CV-AM fluorescence of unstained cells. Mycobacteria were sampled from three replicate steady-state fast-growth (MGT, 23.1 h) or slow-growth (MGT, 69.3 h) chemostat cultures and stained with CV-AM, fixed with formaldehyde, and analyzed by flow cytometry. Fast-growing populations of bacteria stained very brightly with CV-AM, and the three replicates gave median fluorescence values of 300.91, 270.01, and 280.21 (mean fluorescence for all three replicates ± SD, 283.71 ± 15.71) for fast growers and 168.83, 121.98, and 137.03 for slow growers (mean fluorescence for all three replicates ± SD, 142.61 ± 23.91), demonstrating that slow-growing organisms stained less brightly by approximately twofold. The differences between these populations were shown to be statistically significant using a two-tailed *t* test with a *P* value of 0.001.

Cells growing at different rates were distinguishable from each other by the use of fluorescent flow cytometry. The dye CV-AM, which measures esterase activity, was able to differentiate populations of bacteria cultured at different growth rates (Fig. 1). Cells were sampled from three replicate steady-state fast-growth (MGT, 23.1 h; mean cell titer ± standard deviation [SD], 9.2 × 10^7^ CFU ml^−1^ ± 1.9 × 10^6^ CFU ml^−1^) or slow-growth (MGT, 69.3 h; mean cell titer ± SD, 7.42 × 10^7^ CFU ml^−1^ ± 1.6 × 10^6^ CFU ml^−1^) chemostat cultures and stained with CV-AM, fixed with formaldehyde, and analyzed for viability by flow cytometry (27, 28). Fast-growing populations of bacteria stained very brightly with CV-AM and gave median fluorescence values for the three replicates of 300.91, 270.01, and 280.21 (mean fluorescence for all three replicates ± SD, 283.71 ± 15.71), while for slow growers, the median fluorescence values for the three replicates were 168.83, 121.98, and 137.03 (mean fluorescence for all three replicates ± SD, 142.61 ± 23.91), demonstrating that slow-growing organisms stained less brightly by approximately 2-fold (*P* = 0.001) (Fig. 1).

### Sytox green stains dead M. tuberculosis cells.

**FIG 2:**
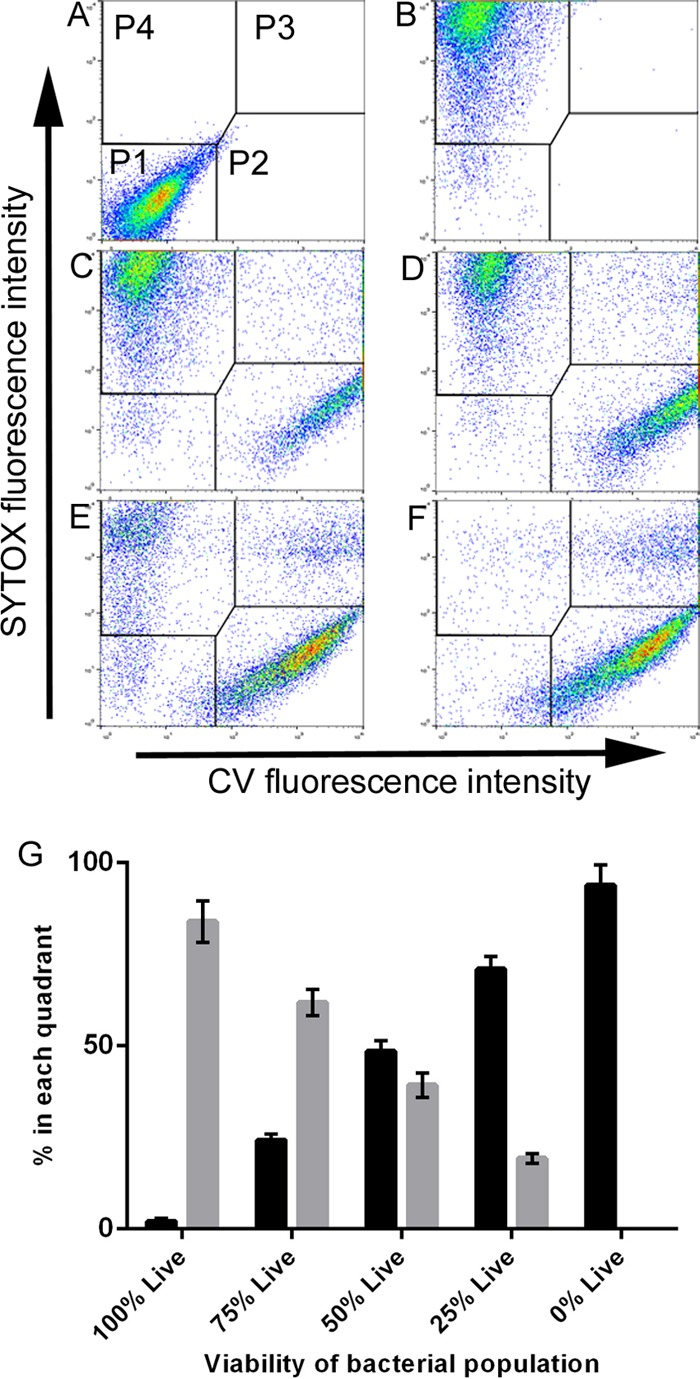
Representative image of dual-parameter dot plots of premixed heat-killed and live cells unstained or dual stained with CV and SG in the following percentage ratios: unstained (live population) (A), 100:0 (B), 75:25 (C), 50:50 (D), 25:75 (E), and 0:100 (F). (G) Average percentage of events (from four independent experiments) in the SG-positive, CV-negative population (P4, black bars) and CV-positive, SG-negative population (P2, gray bars). Population gates P1 to P4 are indicated in panel A; a full description of these gates can be found in Table 1. The *x* and *y* axes represent the increase in fluorescence intensity. The scales are logarithmic and range incrementally from 0 to 10^4^.

We mixed populations of heat-killed cells and chemostat-grown exponential-phase cells (MGT, 23.1 h) in the following ratios: 100:0, 75:25, 50:50, 25:75, and 0:100. These premixed cells were dual stained with SG and CV-AM and then analyzed by flow cytometry. This was repeated on four separate occasions with samples from four independent chemostats. The mean average percentage of cells in the live and dead population gates of the dual-parameter plots from the four experiments reflected the starting percentages of heat-killed cells and live cells in the premixed populations (Fig. 2). The Pearson product moment correlation coefficient (*r*) for all four replicates was 0.99.

### Antibiotic susceptibility.

M. tuberculosis cell samples from the fast-growth chemostats (MGT, 23.1 h) were exposed to either rifampin or isoniazid over a range of concentrations, including the MIC (0.5 μg ml^−1^ and 32 ng ml^−1^ for isoniazid and rifampin, respectively) and up to 16× MIC, over a time course and incubated at 37°C in shaking (200 rpm), aerated broth cultures. The bacilli were initially exposed to eight antibiotic concentrations during preliminary 4-day drug exposure experiments to enable a working set of concentrations to be refined. Cells were sampled every 24 h, dual stained with CV-AM and SG, and analyzed by either flow cytometry or the method of Miles et al. (29). Populations of cells were gated depending on the positions of the controls within the two-parameter dot plot. Considering the known properties of the two dyes and our confirmation of their activities, as shown in Fig. 1 and 2 (CV-AM staining of bacteria at different growth rates and SG staining of heat-killed cells), we interpreted maximum SG staining to be indicative of a dead population. Likewise, we interpreted CV-AM staining to be indicative of a viable population, which is defined in this case as cells that possess esterase activity, and SG and CV-AM dual staining to be indicative of a viable but cell wall-compromised population. Each population gate (gates P1 to P4) of a dual-parameter plot represents a different population of stained cells: gate P1 represents cells that did not stain with either CV-AM or SG, gate P2 represents cells that stained maximally with CV-AM, gate P3 represents cells that stained maximally with CV-AM and SG, and gate P4 represents cells that stained maximally with SG (see Fig. 2A for an explanation of the gate layout of a dual-parameter plot). The sterilizing action of rifampin and isoniazid would be expected to result in a majority CV-AM-stained population in gate P2 at day 0 that would then migrate via a dual-stained population in gate P3 to a majority SG-stained population in gate P4.

**FIG 3:**
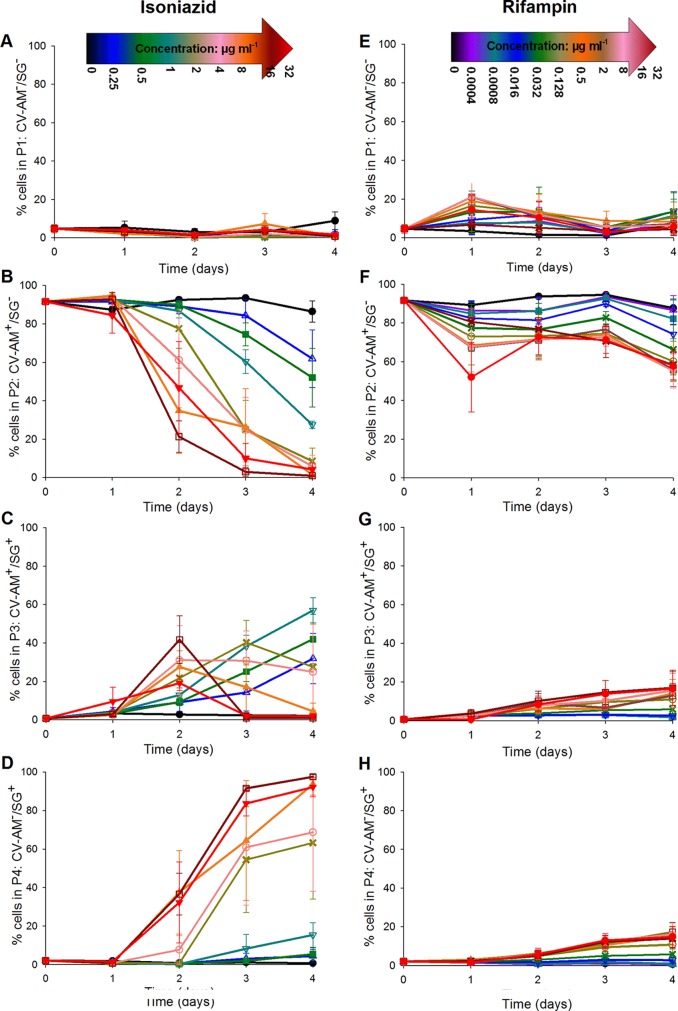
Average percentage of the mycobacterial cell population that was either not fluorescently labeled (A and E), labeled with CV-AM only (B and F), labeled with both CV-AM and SG (C and G), or labeled with SG only (D and H). Cell samples from three independent fast-growth chemostat cultures were exposed to selected concentrations of either isoniazid (A to D) or rifampin (E to H) over a 4-day time course, followed by dual staining with CV-AM and SG and analysis by flow cytometry. Symbols for isoniazid concentrations: ● (black), 0 μg ml^−1^; △ (blue), 0.25 μg ml^−1^; ■ (green), 0.5 μg ml^−1^; ▽ (cyan), 1 μg ml^−1^; × (buff), 2 μg ml^−1^; ○ (pink), 4 μg ml^−1^; ▲ (orange), 8 μg ml^−1^; □ (dark red), 16 μg ml^−1^; ▼ (red), 32 μg ml^−1^. Symbols for rifampin concentrations: ● (black), 0 μg ml^−1^; △ (purple), 0.004 μg ml^−1^; ■ (cyan), 0.008 μg ml^−1^; ▽ (blue), 0.016 μg ml^−1^; × (green), 0.032 μg ml^−1^; ○ (buff), 0.128 μg ml^−1^; ▲ (orange), 0.5 μg ml^−1^; □ (brown), 2 μg ml^−1^; ▼ (pink), 8 μg ml^−1^; × (dark red), 16 μg ml^−1^; ● (red), 32 μg ml^−1^. Error bars represent standard errors of the means (*n =* 3). CV-AM^−^, not labeled with CV-AM; SG^−^, not labeled with SG; CV-AM^+^, labeled with CV-AM; SG^+^, labeled with SG.

The proportions of bacteria in each population gate were averaged across three independent biological replicates for each antibiotic and were expressed graphically as a percentage of the whole population (Fig. 3). At zero antibiotic levels, the percentage of cells that stained with SG only (gate P4) or CV-AM only (gate P2) remained steady at about 0 to 5% (Fig. 3D and H) or 90 to 95% (Fig. 3B and F), respectively, showing that after 4 days the cells were still viable in the absence of antibiotic. Approximately 5% of the bacterial population steadily shifted from gate P2 to gate P1 (Fig. 3A and E) over the 4-day time course. This could be because a proportion of the bacterial population naturally entered the start of stationary phase due to nutrient limitation (Fig. 3). The potential for fluorescence shifts in the presence of the antibiotics (particularly rifampin) was tested by examining unstained populations exposed to antibiotic (at all concentrations) at day 0. The results showed that no increase in the autofluorescence was observed at any concentration of isoniazid (0 to 32 μg ml^−1^) or rifampin (0 to 32 μg ml^−1^) tested (see Fig. S1 in the supplemental material).

### Isoniazid exposure.

**FIG 4:**
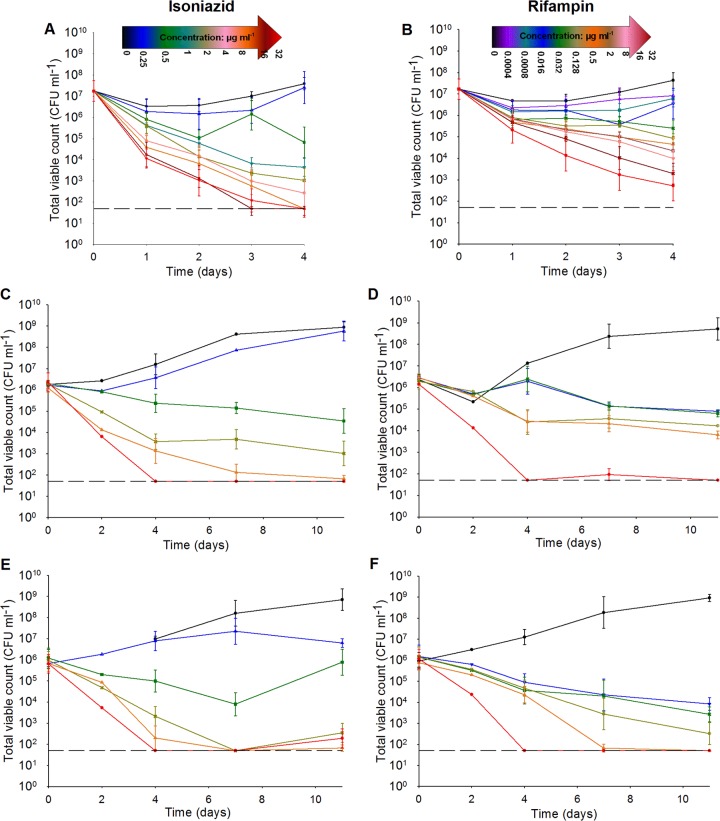
Average viable counts (number of CFU milliliter^−1^) of cells from three different antibiotic exposure experiments. (A and B) Cell samples from three independent fast-growth chemostat cultures were exposed to selected concentrations of either isoniazid (A) or rifampin (B) over a 4-day time course. (C and D) Cells were also exposed to isoniazid (C) or rifampin (D) for 4 days before being resuspended in antibiotic-free medium for a further 7 days. (E and F) An extended time course of treatment with isoniazid (E) or rifampin (F) over 11 days was also performed. Samples were taken at various time points throughout the experiments, serially diluted, and plated by the method of Miles et al. (29). Symbols for isoniazid concentrations: ● (black), 0 μg ml^−1^; △ (blue), 0.25 μg ml^−1^; ■ (green), 0.5 μg ml^−1^; ▽ (cyan), 1 μg ml^−1^; × (buff), 2 μg ml^−1^; ○ (pink), 4 μg ml^−1^; ▲ (orange), 8 μg ml^−1^; □ (dark red), 16 μg ml^−1^; ▼ (red), 32 μg ml^−1^. Symbols for rifampin concentrations: ● (black), 0 μg ml^−1^; △ (purple), 0.004 μg ml^−1^; ■ (cyan), 0.008 μg ml^−1^; ▽ (blue), 0.016 μg ml^−1^; × (green), 0.032 μg ml^−1^; ○ (buff), 0.128 μg ml^−1^; ▲ (orange), 0.5 μg ml^−1^; □ (brown), 2 μg ml^−1^; ▼ (pink), 8 μg ml^−1^; × (dark red), 16 μg ml^−1^; ● (red), 32 μg ml^−1^. Error bars represent relative standard errors (*n =* 3).

At the MIC level of isoniazid (0.5 μg ml^−1^), a reduction of the viable counts of almost 2 log_10_ CFU ml^−1^ (Fig. 4A) was observed by day 4, and this translated to a 40% reduction in the CV-AM-stained population (Fig. 3B, gate P2, no SG staining). This was also accompanied by an increase in the counts of cells that were maximally stained with both CV-AM and SG; these cells are defined as showing signs of cell wall damage through antibiotic action (Fig. 3C, gate P3). At this MIC level of isoniazid, there was only a slight increase in the percentage of cells that stained with SG alone (Fig. 3D, gate P4, no CV-AM staining), and this result was not statistically significantly different from that obtained with 0 μg ml^−1^ (*P* = 0.177, Mann-Whitney U test). These data show inhibition of cells but no apparent increase in cell death (as defined earlier). The 2-log_10_ CFU ml^−1^ reduction in colonies could be reflective of organisms that remained viable but that were not immediately culturable under our *in vitro* conditions. The bactericidal effect of isoniazid was observed at an isoniazid concentration of 2 μg ml^−1^ (4× MIC); the viable counts were reduced by 4 log_10_ CFU ml^−1^ (99.9%) at day 4 (Fig. 4A). According to the results of the flow cytometry analyses, only 60% of this reduced population consisted of cells that were specifically stained with SG (Fig. 3D, gate P4). An additional 30% of the reduction in the viable population, observed by plating by the method of Miles et al. (29), could potentially be identified by staining with SG and CV-AM (Fig. 3C, gate P3, cells were damaged but still produced esterases that would cleave CV-AM). The presence of this dual-stained population indicated that the flow cytometry analyses might be able to distinguish between cells that were dead and cells that were damaged, unlike the plating method, which revealed information only about bacteria that could be further propagated on agar postantibiotic exposure. At the high isoniazid concentrations of 8 μg ml^−1^ and 32 μg ml^−1^, a bactericidal effect was observed by day 2, and by day 4 the CV-AM-stained population was reduced by more than 90% (Fig. 3B and D, gates P3 and P2) (*P* = 0.032, Kruskal-Wallis test). In accordance with the decrease in the CV-AM-stained population, the SG-stained population increased to nearly 100% by day 4 (Fig. 3D, gate P4) at the highest isoniazid concentration tested of 32 μg ml^−1^, resulting in complete killing (limit of detection, 50 CFU ml^−1^) (Fig. 4A).

### Rifampin exposure.

The results of rifampin exposure were in stark contrast to those of isoniazid exposure, as shown by the difference in the changing staining patterns of cells exposed to the two antibiotics in the dual-parameter plots (Fig. 3; see also Fig. S2 in the supplemental material).

By day 4, at all concentrations of rifampin tested, at least 70% of the total population was stained with CV-AM (Fig. 3F and G, gates P3 and P2), with no significant difference between the concentrations tested being detected (*P* = 0.153, Kruskal-Wallis test). In contrast, at all concentrations tested only up to 30% of the total population was stained with SG (Fig. 3G and H, gates P4 and P3). There was some suggestion of a concentration-dependent response over time, but it was not to the same extent seen with the isoniazid-treated cells (Fig. 3D), as highlighted by the fact that at the highest concentration of rifampin tested (32 μg ml^−1^), only 30% of the population was either dead or damaged (Fig. 3G and H). Unlike cells treated with a high concentration of isoniazid (which were maximally stained with SG and not CV-AM), approximately 20% of the cells treated with a high concentration of rifampin were stained with SG and CV-AM (Fig. 3G, gate P3), indicating inhibition of growth and possible cell damage. This lack of sterilization was consistently observed in the viable counts; by day 4, the highest concentration of 32 μg ml^−1^ tested gave a 4-log_10_ CFU ml^−1^ reduction in bacterial counts, leaving a viable population of about 0.1% of the original population that was culturable on plates (Fig. 4B).

### Time-dependent sterilization with rifampin.

The 4-day drug exposure indicated that the rifampin-treated population was not sterilized to the same extent as the isoniazid-treated population by day 4 and suggested that the sterilizing activity of rifampin against M. tuberculosis was more time dependent than that of isoniazid. To address this, modified drug exposure experiments were performed.

**FIG 5:**
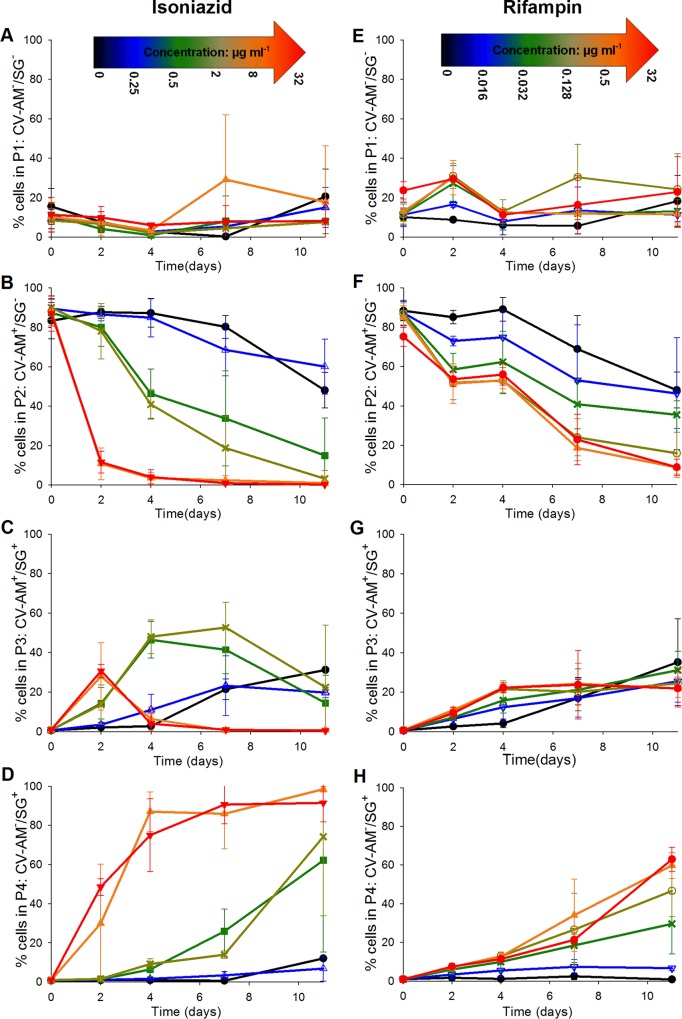
Average percentage of the mycobacterial cell population that was either not fluorescently labeled (A and E), labeled with CV-AM only (B and F), labeled with both CV-AM and SG (C and G), or labeled with SG only (D and H). Cell samples from three independent fast-growth chemostat cultures were exposed to selected concentrations of either isoniazid (A to D) or rifampin (E to H) over a 4-day time course. The antibiotic was removed by resuspending the cells in fresh medium, and the cells were incubated for a further 7 days in the absence of antibiotic. This was followed by dual staining with CV-AM and SG and analysis by flow cytometry and viable count analyses, as described in the legend to Fig. 4. Symbols for isoniazid concentrations: ● (black), 0 μg ml^−1^; △ (blue), 0.25 μg ml^−1^; ■ (green), 0.5 μg ml^−1^; × (buff), 2 μg ml^−1^; ▲ (orange), 8 μg ml^−1^; ▼ (red), 32 μg ml^−1^. Symbols for rifampin concentrations: ● (black), 0 μg ml^−1^; ▽ (blue), 0.016 μg ml^−1^; × (green), 0.032 μg ml^−1^; ○ (buff), 0.128 μg ml^−1^; ▲ (orange), 0.5 μg ml^−1^; ● (red), 32 μg ml^−1^. Error bars represent standard errors of the means (*n =* 3).

Further 4-day exposure time courses (three replicates) were performed, followed by resuspension in fresh growth medium. The bacterial cultures were then incubated for a further 7 days in the absence of antibiotic; flow cytometry analyses and total viable counts were performed as described above. Growth in a zero level of antibiotic resulted in an increase in the viable count to 9 log_10_ CFU ml^−1^ over 11 days (Fig. 4C and D), with a plateau in the CV-AM-stained population at about 70 to 80% of the original population occurring at 3 days postresuspension (Fig. 5B and F, gate P2). By day 11, the CV-AM stained population in gate P2 was reduced to ∼60%, which was accompanied by a rise in the population in gate P3 dual stained with SG and CV-AM (Fig. 5C and G). This could be an early indication of a slowing in the growth rate toward the end of the culture time course, which would not be detected as early by viable counts. The population of SG-stained bacteria in gate P4 remained steady at ∼5% throughout the time course, indicating no increase in the number of dead bacilli (Fig. 5D and H).

For rifampin-treated bacteria, the removal of antibiotic after 4 days of incubation was followed by continued bactericidal responses at days 5 to 11 at all concentrations of antibiotic tested. The bacilli exhibited a concentration-dependent response postresuspension following exposure to high doses, 0.5 μg ml^−1^ and 32 μg ml^−1^, resulting in the most marked bactericidal response. At these high concentrations at day 11, ≤10% of the population was stained singly with CV-AM (Fig. 5F, gate P2) and ∼60% was stained singly with SG (Fig. 5H, gate P4). The remaining population in gate P3 had plateaued at ∼20% (Fig. 5G). The accompanying total viable count analyses revealed that only the highest concentration of rifampin tested resulted in a complete absence of colonies from day 4 onwards. At a concentration of 0.5 μg ml^−1^, the viable population was about 3 log_10_ CFU ml^−1^ after 11 days of exposure (Fig. 4D), suggesting that, despite removal of the antibiotic at day 4, rifampin exhibited concentration-dependent postantibiotic effects which at concentrations as high as 0.5 μg ml^−1^ did not lead to sterilization. Isoniazid showed a similar postantibiotic effect at lower relative concentrations of about 0.5 μg ml^−1^ and 2 μg ml^−1^ (the MIC and 4× MIC, respectively). However, as supported by the findings with the initial 4-day exposure, high concentrations of isoniazid resulted in approximately 90% of the population being stained with SG by day 4, followed by a plateauing at this level to day 11 (Fig. 5D). These findings suggest that rifampin has more of a time-dependent killing mode of action than isoniazid.

**FIG 6:**
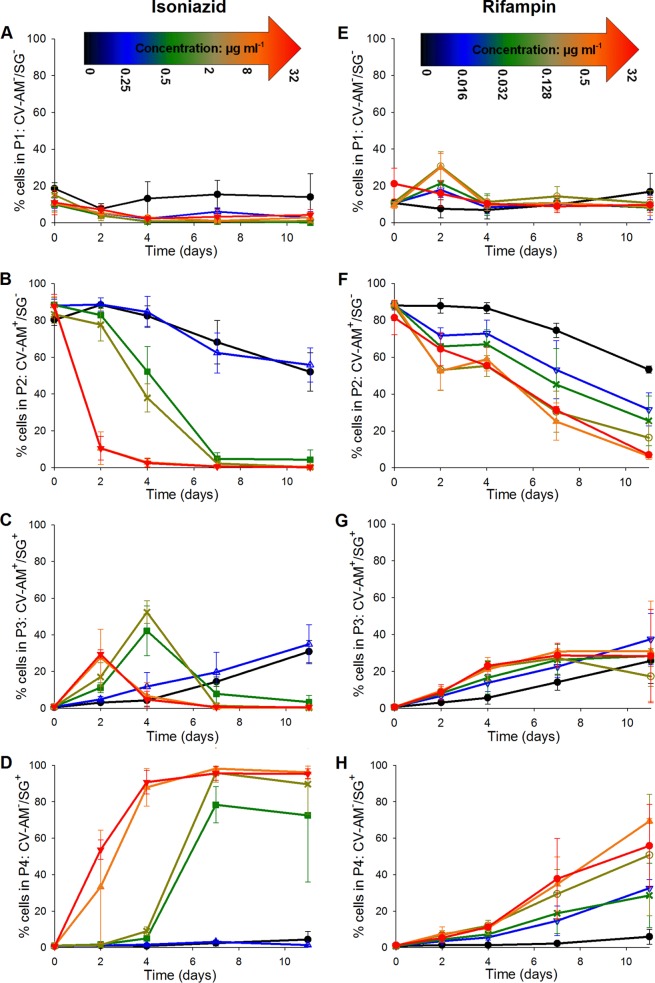
Average percentage of the mycobacterial cell population that was either not fluorescently labeled (A and E), labeled with CV-AM only (B and F), labeled with both CV-AM and SG (C and G), or labeled with SG only (D and H). Cell samples from three independent fast-growth chemostat cultures were exposed to selected concentrations of either isoniazid (A to D) or rifampin (E to H) over an 11-day time course. This was followed by dual staining with CV-AM and SG and analyses by flow cytometry and viable count analyses, as described in the legend to Fig. 4. Symbols for isoniazid concentrations: ● (black), 0 μg ml^−1^; △ (blue), 0.25 μg ml^−1^; ■ (green), 0.5 μg ml^−1^; × (buff), 2 μg ml^−1^; ▲ (orange), 8 μg ml^−1^; ▼ (red), 32 μg ml^−1^. Symbols for rifampin concentrations: ● (black), 0 μg ml^−1^; ▽ (blue), 0.016 μg ml^−1^; × (green), 0.032 μg ml^−1^; ○ (buff), 0.128 μg ml^−1^; ▲ (orange), 0.5 μg ml^−1^; ● (red), 32 μg ml^−1^. Error bars represent standard errors of the means (*n =* 3).

Given these findings, antibiotic exposure was extended to 11 days to assess whether the characteristic movement in the majority stained population from gate P2 to gate P4 observed for isoniazid-sterilized cells at day 4 could also be observed for rifampin exposure but later in the time course (Fig. 6). The variability between replicate time courses for each population gate and for each antibiotic tested was assessed and found to be low. For cultures exposed to isoniazid, the coefficients of variance (CoVs) were 12.9% (gate P1), 13.1% (gate P2), 9.4% (gate P3), and 8.5% (gate P4). For cultures exposed to rifampin, the CoVs were 33.3% (gate P1), 18.5% (gate P2), 2.9% (gate P3), and 3.9% (gate P4). During the extended time courses, at a zero concentration of antibiotic, there was a gradual increase in the number of colonies after day 4 in culture, which continued to rise to 9 log_10_ CFU ml^−1^ by day 7 and eventually plateaued at this cell titer by day 11 (Fig. 4E and F). However, there was a steady shift in 20% of the CV-AM-stained bacterial population from gate P2 to gate P3 between days 4 and 7, and by day 11, there was a further shift of 10% of the population (Fig. 6B, C, F, and G). This was unexpected, as cells were still dividing, as confirmed by the colony counts. The CV-AM staining was performed immediately after sampling from the culture and was therefore possibly more indicative of bacterial health than the viable counts, which were calculated from colonies that were provided with fresh nutrients in agar plates and given 3 weeks of incubation to become visible. The CV-AM staining could provide alternative (and crude) information about the phenotype or growth rate in culture.

At sub-MIC levels of isoniazid (0.25 μg ml^−1^), the viable count plateaued at about 7 log_10_ CFU ml^−1^ between day 4 and day 11 (Fig. 4E), and the CV-AM staining profile was very similar to that achieved with 0 μg ml^−1^ isoniazid, indicating that exposure to a sub-MIC level of isoniazid had no significant additional effect over nutrient depletion in culture (*P* = 0.996, *t* test) (Fig. 6B). Isoniazid at 0.5 μg ml^−1^, 2 μg ml^−1^, 8 μg ml^−1^, and 32 μg ml^−1^ resulted in a bactericidal response within the first 4 days. This was followed by a further reduction in the cell number to 4 log_10_ CFU ml^−1^ by day 7 for the MIC level of isoniazid (Fig. 4E), which was reflected by an increase in SG staining in gate P4 (Fig. 6D). Interestingly, at this concentration there was a rebound in the viable count to 6 log_10_ CFU ml^−1^ by day 11 (Fig. 4E), which has been observed in previous studies (34–36). As expected from the initial 4-day exposure experiments, high concentrations of the antibiotic resulted in no colonies, and approximately 90% of the population was stained with SG by day 4, followed by a plateauing at this level to day 11 (Fig. 6D). CV-AM staining showed a clearer concentration-dependent response than the viable counts, which could be reflective of different growth rates in the population and will be useful for separating out different subpopulations for further analyses. The concentration-dependent response was also reflected in the SG-stained population in gate P4. By day 11, 70% of the population exposed to 0.5 μg ml^−1^ (the MIC) was in gate P4, at 2 μg ml^−1^ the population was at about 90%, and at 8 μg ml^−1^ and 32 μg ml^−1^, the percentage in gate P4 continued to plateau at about 95% (Fig. 6D).

The primary purpose for the extended time course was to see whether the characteristic shift from gate P2 to gate P4 could be achieved and determine whether rifampin required a longer time course to achieve sterilization. Over the 11-day time course, 0.5 μg ml^−1^ and 32 μg ml^−1^ (16× MIC and 1,000× MIC, respectively) achieved total killing by day 11, as determined by viable count analyses (Fig. 4F). This was reflected by a CV-AM-stained population percentage of ≤10% in gate P2 and an ∼70% SG-stained population in gate P4 (Fig. 6F and H). The CV-AM-stained population was significantly lower at day 11 than at day 4 at all concentrations of rifampin, and the SG-stained population significantly increased after day 11 compared with that obtained by incubation for 4 days at four out of the six concentrations tested (*P* < 0.05, two-way analysis of variance [ANOVA] with Holm-Sidak *post hoc* testing). Lower levels of rifampin resulted in a dose-response (*P* < 0.001, two-way ANOVA) as well as a time-dependent response (*P* < 0.001, two-way ANOVA) in both the total viable counts (Fig. 4F) and the CV-AM and SG staining profiles (Fig. 6F and H).

### Dual-stained fluorescence profiles are indicative of antibiotic mode of action.

Preliminary experiments suggested that the different patterns of fluorescence profiling for rifampin and isoniazid reflected a difference in their modes of action. This was explored further by analyzing the fluorescence profiles of bacilli that had been treated with additional antibiotics that possessed a range of modes of action.

**FIG 7:**
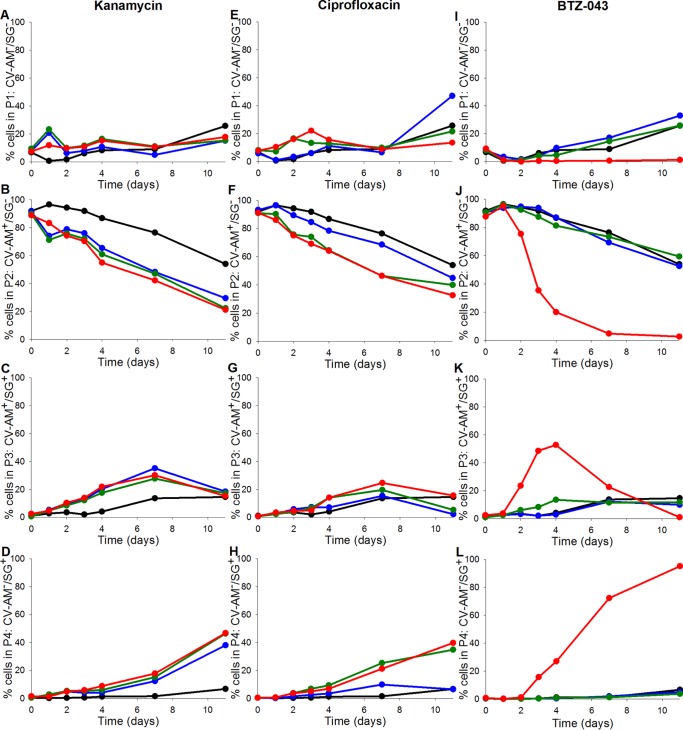
Percentage of the mycobacterial cell population that was either not fluorescently labeled (A, E, and I), labeled with CV-AM only (B, F, and J), labeled with both CV-AM and SG (C, G, and K), or labeled with SG only (D, H, and L). Cell samples from fast-growth chemostat cultures were exposed to selected concentrations (equivalent to 0.25×, 1×, and 4× published MIC) of either kanamycin (A to D), ciprofloxacin (E to H), or BTZ-043 (I to L) over an 11-day time course, followed by dual staining with CV-AM and SG and analyses by flow cytometry. Colors of circles for kanamycin concentrations: black, 0 μg ml^−1^; blue, 0.5 μg ml^−1^; green, 2 μg ml^−1^; red, 8 μg ml^−1^. Colors of circles for ciprofloxacin concentrations: black, 0 μg ml^−1^; blue, 0.125 μg ml^−1^; green, 0.5 μg ml^−1^; red, 2 μg ml^−1^. Colors of circles for BTZ-043 concentrations: black, 0 μg ml^−1^; blue, 0.0007 μg ml^−1^; green, 0.0015 μg ml^−1^; red, 0.006 μg ml^−1^. The data are from a single experiment.

Three further bactericidal antibiotics were selected on the basis of their mode of action; two had an intracellular target (kanamycin and ciprofloxacin), and one had a cell wall target (BTZ-043). They were tested according to the 11-day exposure method as described above at 0.25× MIC, the MIC, and 4× MIC (Fig. 7).

Qualitative observations indicated that the intracellularly targeted and cell wall-targeted antibiotics could be distinguished on the basis of their fluorescence profiles. For example, the early and steep rise in SG staining in gate P4 by day 4 and the characteristic peak of fluorescence in gate P3 by day 2 could be observed in studies with both the cell wall-active antibiotics, isoniazid and BTZ-043 (Fig. 6C and D and 7K and L). Similarly, the steady rise in the SG-stained population observed in gate P4 with rifampin exposure was also observed with all the other intracellularly targeting antibiotics (Fig. 6H and 7D and H). The two different groups of antibiotics also demonstrated consistent spikes in their fluorescent profiles. During exposure to intracellularly targeting antibiotics, the population in gate P3 consistently spiked at about day 7 at all concentrations tested. There was a similar but more striking peak of fluorescence in gate P3 at day 4 that persisted until about day 7 for all the cell wall-targeting antibiotics. With isoniazid-treated cultures, for which a wider range of concentrations was used, this spike in gate P3 was dependent on the concentration with the highest concentrations causing an earlier spike at day 2 (Fig. 6C).

In addition to similarities in the fluorescence profiles for antibiotics with an equivalent mode of action, the fluorescence labeling also highlighted differences in the organism's responses to the different antibiotics. The organism showed a clearer concentration-dependent response to ciprofloxacin in terms of the increasing proportion of SG-stained populations as the antibiotic concentration increased (Fig. 7G and H). In contrast, kanamycin treatment resulted in a 50% SG-stained population with all three concentrations tested (Fig. 7C and D). The trend in the SG-stained population achieved with BTZ-043 was very similar to that achieved with isoniazid. BTZ-043 at 0.25× MIC and the MIC did not show any signs of killing, whereas the organism showed a clear bactericidal response to 4× MIC of BTZ-043 that was distinct from that to the two lower concentrations, as demonstrated by 95% of the population being SG stained in gate P4 (Fig. 7L).

## DISCUSSION

### Calcein violet-AM and Sytox green staining of M. tuberculosis.

Calcein-AM (in this case, not calcein violet) has previously been used for determining drug toxicity and drug activity in mammalian systems but not for determining bacterial viability (37–39). However, a combination of CV-AM and Sytox red has been compared with other dye pairs for their ability to visualize and quantify live/dead populations during the first phase of bioadhesion in the formation of oral bacterial biofilms (11), and more recently, it has been shown that CV fluorescence is a direct measure of metabolic activity in Corynebacterium glutamicum (12).

Modification of CV with an acetoxy-methyl ester (AM) group results in an uncharged molecule that can permeate cell membranes. Once inside the cell, the lipophilic blocking groups are cleaved by nonspecific esterases, resulting in a charged form that is retained in cells to a much greater extent than its parent compound. We selected CV-AM for its potential in discriminating between populations of M. tuberculosis cells in particular states that are thought to predispose the organism to the development of drug-tolerant persistence, such as slow growth.

CV-AM in combination with flow cytometry has potential utility to rapidly determine the activity of antibiotics that have previously been studied for their efficacy against M. tuberculosis in relevant phenotypic states (34, 40–43). Parallels between our study and a study by Tawakoli et al. (11) can be drawn, in that more traditional plating methods were unable to quantify viable but not yet culturable oral bacteria; using current approaches, over 50% of the oral bacterial microbiota could not be cultured, and the use of CV-AM staining allowed a clear distinction between the different susceptibility phenotypes within the biofilms (44, 45). The unculturable organisms (Synergistetes) have since been grown in the presence of other bacteria, providing further evidence of the specific growth requirements of VBNC microorganisms (46).

Through direct measurement of esterase levels, we found that slow-growing organisms consistently exhibited less fluorescence (by approximately 2-fold) than fast-growing cells, enabling bacteria at different growth rates to be distinguished using CV-AM staining (Fig. 1). It is also possible that differential CV-AM staining is not due to changes in metabolic activity *per se* but, rather, is due to variations in uptake or efflux due to differences in the growth rates of the two populations. One way of determining this would be to measure the intracellular levels of dye that has not yet reacted with esterase. However, this would be technically challenging, as CV-AM remains colorless and nonfluorescent until it has been hydrolyzed. Irrespective of the reasons why the slow-growing and fast-growing bacteria fluoresce at different levels, the critical point is that this difference is consistent and that is the primary parameter required for the flow method to be utilized.

Syto 9 and propidium iodide (PI) dyes were previously tested by Soejima et al. (47) using the avirulent strain M. tuberculosis H37Ra and were found to be effective in discriminating live M. tuberculosis bacteria from antibiotic-injured or dead bacteria. Distinction between these subpopulations could be made only with the additional step of ethidium monoazide (EMA) treatment and light visualization. EMA has the disadvantage of being irreversibly bound to the DNA, which leads to cell death. In the study described by Tawakoli et al. (11), CV-AM and Sytox red dual staining performed the best in terms of cell viability poststaining; at least 50% of the bacterial population was still viable. The approach that we describe here is simpler, as it does not require the use of EMA and light visualization; we are also aiming for a viable population poststaining for downstream analyses. We chose SG for its specificity of staining dead bacteria. Unlike SG, the action of the Syto dyes is nonspecific, as they permeate and label all bacterial cells to some degree. We have demonstrated that SG reproducibly stains heat-killed bacteria and also stains bacteria that have been treated with high concentrations of antibiotic.

The use of single dyes has previously been shown to be applicable for identifying drug-resistant bacteria. FDA was one of the first dyes to be used that could assess the viability of bacteria, and more recently, further studies have successfully combined the use of FDA with microscopy to rapidly detect MDR M. tuberculosis bacteria in sputum (48). Like CV-AM, FDA is a dye that is hydrolyzed by cytosolic esterases. FDA accumulates inside viable bacteria, while bacteria that are inhibited by antibiotics exhibit reduced hydrolysis of FDA and reduced fluorescence. However, unlike CV, which is stable, FDA can rapidly leak out of cells, resulting in a lack of fluorescence by bacteria that are viable (14). A single staining approach has limitations in differentiating between viable bacteria with low metabolic activity and bacteria that are dead.

Unlike the study by Tawakoli et al. (11), who used microscopy to visualize fluorescently stained bacteria, the use of flow cytometry enabled us to gate and enumerate each subpopulation with a view to collecting these populations for further characterization while retaining the ability to observe the heterogeneity within a population. The study by Soejima et al. (47) used an avirulent strain of M. tuberculosis and, therefore, did not tackle the technical and practical challenges of flow cytometry analyses of an Advisory Committee for Dangerous Pathogens hazard group 3 organism (ACDP3; equivalent to biosafety level 3 [BSL3]). PI, Syto 9, and FDA are not fixable dyes; fixation of M. tuberculosis with formaldehyde for a length of time sufficient to ensure cell death while retaining fluorescence needed to be considered here. In contrast, CV-AM and SG staining is fixable, allowing removal of stained cells from the high-containment environment for flow analyses, which enables the implementation of this method in resource-constrained clinical settings where high-containment flow cytometry facilities are too expensive or not available.

### Antibiotic susceptibility.

Traditional antibiotic susceptibility methods inform on the bactericidal effect of the drug by enumerating bacteria that can be cultured. The purpose of developing a fluorescence flow method using the dyes CV-AM and SG was to complement current susceptibility testing methods by providing information about all subpopulations while also providing a means of isolating them for further study in the future. While it was important for validation of the flow method to show similar trends in the susceptibility profiles between the fluorescence profiling and the viable count analyses, it was not essential that the two methods be exactly comparable. The purpose of the flow method is to observe the entire population, whereas plate counting is an indirect measure of sterilization, quantifies only viable bacteria that are culturable on agar, and generates no information on organisms that are not immediately culturable under standard *in vitro* conditions as a result of antibiotic exposure (6, 49, 50). Exposure to the MIC level of isoniazid for 4 days resulted in a loss of 2 log_10_ CFU ml^−1^ of viable colonies. If the loss of all viable colonies was a result of cell death, then it appears that the SG staining profiles do not reflect the bactericidal response. However, if, as we predicted on the basis of previous findings, that there was a subpopulation that was not immediately culturable, then the flow method could provide a more realistic representation of the whole population of cells. Not only can different subpopulations be visualized by the flow method, but also they can be gated independently and collected using a contained cell sorter for further phenotypic and genotypic characterization.

### Time-dependent killing by rifampin.

The fluorescence profiles followed trends similar to those for the viable count analyses in terms of drug susceptibility, particularly at lower concentrations of rifampin. Both the viable counts and the staining profiles revealed a lack of complete sterilization after 4 days of exposure to rifampin, with the highest rifampin concentration tested (1,000× MIC) still resulting in a population of 10^3^ CFU ml^−1^ cells after 4 days of exposure. We performed further experiments to see if the population in gate P4 (after 4 days of antibiotic exposure) was dead. It has previously been shown that exposure of Saccharomyces cerevisiae to a range of different stresses led to a subpopulation of cells that were apparently dead, as indicated by the uptake of PI. Following a short incubation in the absence of a range of stresses, repair of the membrane damage occurred such that subsequent exposure to PI did not result in staining (51). In the work presented here, cells were resuspended in fresh growth medium for a further 7 days of incubation to see if there would be a shift from gate P4 back to gate P2, indicating regrowth. What we observed was a viable count that plateaued at all concentrations of isoniazid and rifampin tested with a consistent drop in CV-AM fluorescence over time accompanied by a rise in SG fluorescence, the extent of which was linearly concentration dependent. This postantibiotic effect with M. tuberculosis has previously been observed *in vitro* with rifampin (52) in a study that followed methods of resuspension in fresh growth medium similar to those described here.

Even though they reflect the findings of other studies, the results of the experiments described here did not bring us any nearer to determining if the dual stains could be used to give a readout for rifampin-sterilized populations of M. tuberculosis. The incomplete killing after day 4 for rifampin exposure indicated that the sterilizing activity of this antibiotic against M. tuberculosis was more time dependent than that of isoniazid. At high concentrations of rifampin, exposure to the antibiotic for 11 days showed a shift in the stained population from gate P2 to gate P4, as expected for a sterilized population and as seen at earlier time points for isoniazid. Exposure-dependent responses are calculated by multiplying the concentration-dependent effect by the time-dependent effect, and it has previously been shown that both of these parameters for measuring drug response were exhibited by rifampin (34, 53). Our findings were also similar to a pattern of response reported by de Steenwinkel et al. (34) and Jayaram et al. (53, 54), who showed that isoniazid and rifampin differed strongly in their killing rates; isoniazid showed extremely rapid and concentration-dependent killing, whereas rifampin revealed slower, time-dependent killing. In our study, there was a reduction in the bacterial count of 2 log_10_ CFU ml^−1^ and 3 log_10_ CFU ml^−1^ after 3 days of exposure to high concentrations of rifampin of 8 mg liter^−1^ and 16 mg liter^−1^, respectively. These data taken together are consistent with previously published results of clinical studies; early bactericidal activity (EBA) studies showed more rapid killing by isoniazid than rifampin (55, 56).

### Mode of action.

Isoniazid and rifampin have different modes of action. Isoniazid targets mycolic acid biosynthesis in the cell wall, whereas rifampin targets RNA transcription intracellularly. The initial observations that the intracellularly targeting antibiotic profiles and cell wall-targeting drug profiles could be distinguished by their characteristic fluorescence were explored further. Three further bactericidal antibiotics were selected on the basis of their mode of action; these were two intracellularly targeting antibiotics (kanamycin and ciprofloxacin) and a cell wall-targeting antibiotic (BTZ-043). Fluoroquinolones (ciprofloxacin), which target DNA replication (32), have previously shown characteristics of time-dependent killing, whereas aminoglycosides (kanamycin), which target protein synthesis, have demonstrated strong concentration-dependent killing. However, in the study by de Steenwinkel et al. (34), amikacin at a concentration of 256 mg liter^−1^ did not sterilize the population after 3 days of incubation, and sterilization was achieved only after 6 days of incubation, indicating that aminoglycosides also have a time-dependent effect. In our study, we made qualitative observations that the intracellularly targeted and cell wall-targeted antibiotics could be distinguished on the basis of their fluorescence profiles.

The perceived delay in the dual staining of populations that were exposed to intracellularly targeting antibiotics could be because cell wall-targeting antibiotics were disrupting the cell wall more quickly, allowing more SG dye to enter the cells earlier. For this reason, we adopted a dual staining approach using SG and CV-AM. CV-AM is lipophilic and normally permeates the cell membranes of live bacteria. A compromised membrane may also have allowed more CV-AM to pass into the cell, which might have explained the pronounced spike of CV fluorescence seen in the population in the P3 gate (for cells treated with isoniazid or BTZ-043), if it was not for the fact that CV-AM can fluoresce only once the AM group is cleaved off. The level of fluorescence would be influenced by the level of esterase and not the level of the CV-AM diffusion, unless passive diffusion was restricted in some way. Our findings show that the pattern of CV fluorescence is mirrored by the pattern of SG fluorescence, confirming a bactericidal response. There are no previously published findings about the relationship between cell wall integrity and esterase activity for M. tuberculosis. Similar studies using other bacteria showed that a loss of esterase activity could occur rapidly and independently of a loss of membrane integrity (57). If there was a more rapid loss of esterase in isoniazid-treated cells because of differences between the two drugs in the damage caused to the membrane, then we would expect the isoniazid-treated cells to die more rapidly, which is what we observed in both the flow method and the viable counts. Therefore, it would be interesting to know whether there is a relationship between low esterase levels (because esterase has leaked out of a damaged cell) and more rapid cell death. Presumably, if esterase leaked out of a compromised membrane, then so would other cytosolic enzymes required for survival. This would explain the more rapid reduction in bacterial load clinically with isoniazid than rifampin in early bactericidal studies. It seems that the flow method not only tests drug susceptibility but also predicts the antibiotic-specific events that unfold prior to cell death.

### Concluding remarks.

The flow cytometry method described here provides the opportunity to dissect antibiotic-treated populations and characterize subpopulations of cells within them, which will provide new insights into the mechanisms that M. tuberculosis uses to avoid the effects of antibiotic action. The transfer of this technology to resource-constrained clinical settings is feasible, as the CV-AM and SG dyes selected can be fixed inside the bacterial cell, allowing flow cytometry analyses to be performed outside containment facilities. We envisage the assay being further developed as a phenotypic screen for the testing of second-line drugs and defining MDR strains of M. tuberculosis in the absence of reliable genotypic typing of these strains.

This rapid drug susceptibility method may also play a role in identifying more effective antimycobacterials. The differential patterns of fluorescence provided basic information about the mode of action of antibiotics and could therefore provide insights into the likely target of novel drugs. This could accelerate the identification of new treatments of either MDR or drug-tolerant persistent subpopulations, thereby making a contribution to the development of better drug combinations and shorter treatment times for TB.

## Supplementary Material

Supplemental material
