# Supplementary material for: A Flow Cytometry Method for Rapidly Assessing Mycobacterium tuberculosis Responses to Antibiotics with Different Modes of Action
Source: Antimicrob Agents Chemother. 2016 Jun 20;60(7):3869–83. doi: 10.1128/AAC.02712-15 (PMC4914659; doi:10.1128/AAC.02712-15)
Supplement: Supplemental material [file supp_60_7_3869__index.html]

Supplemental material 

# A Flow Cytometry Method for Rapidly Assessing Mycobacterium tuberculosis Responses to Antibiotics with Different Modes of Action

## Supplemental material

- Supplemental file 1 -

  Supplemental Figures S1 and S2

  PDF, 1.3M
