# Supplementary material for: A Flow Cytometry Method for Rapidly Assessing Mycobacterium tuberculosis Responses to Antibiotics with Different Modes of Action
Source: Antimicrob Agents Chemother. 2016 Jun 20;60(7):3869–83. doi: 10.1128/AAC.02712-15 (PMC4914659; doi:10.1128/AAC.02712-15)
Supplement: Supplemental material [file AAC.02712-15_zac005165107so1.pdf]

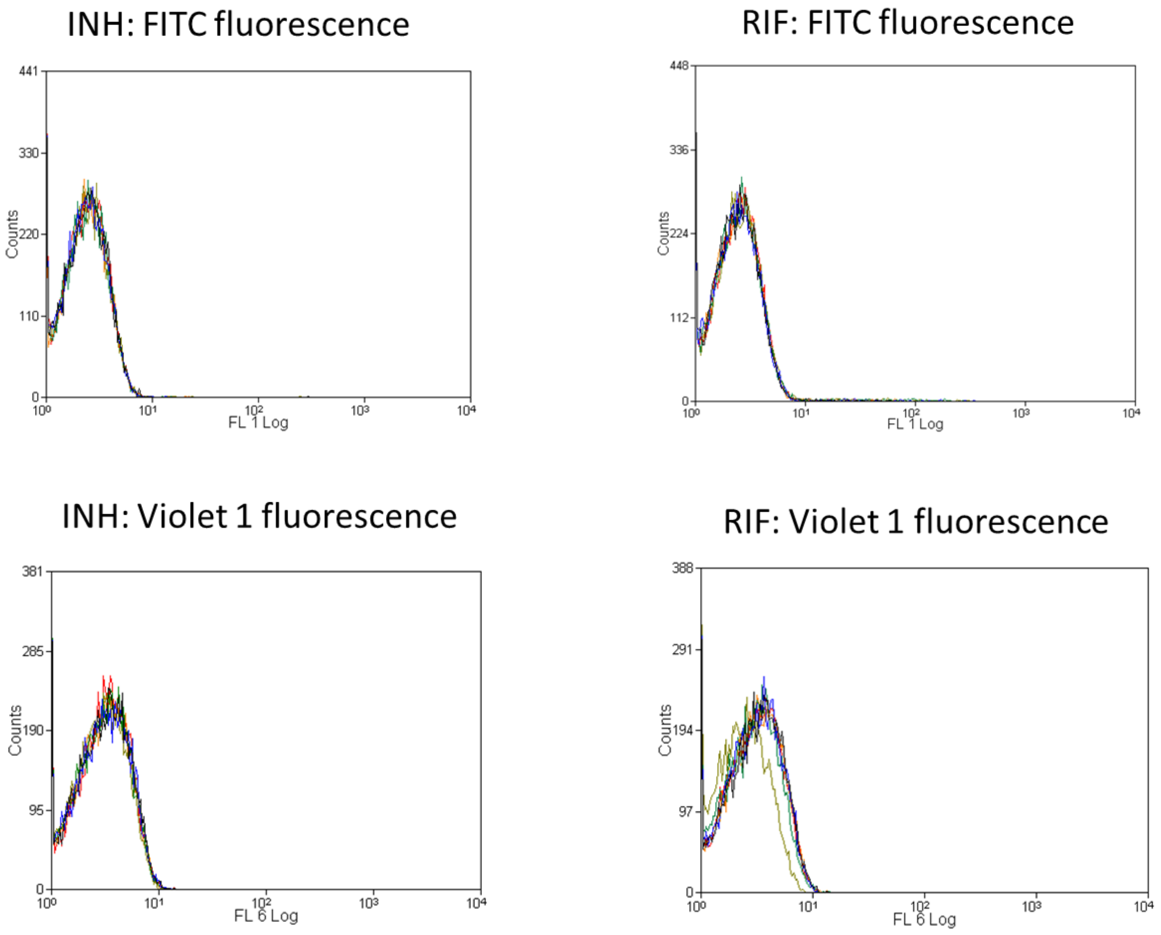

Figure S1 Fluorescence intensities in the FITC channel (SYTOX fluorescence) or in the Violet 1 channel (CV fluorescence), for unstained cell samples of *M. tuberculosis* that have been exposed to either isoniazid (Panels A and C), or rifampicin (Panels B and D). At all concentrations of isoniazid (0 – 32 μgmL^{-1}) or rifampicin (0 – 32 μgmL^{-1}) no increase in the auto-fluorescence was observed.

Isoniazid				
	Day 1	Day 2	Day 3	Day 4
No Drug	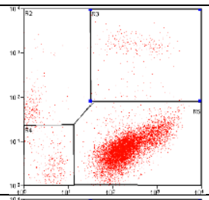	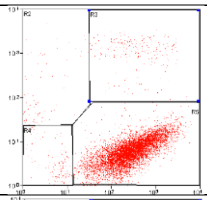	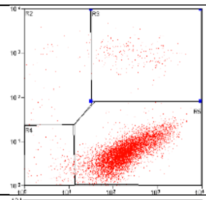	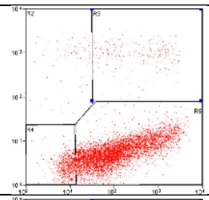
0.25 µg/mL	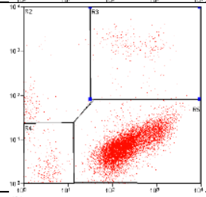	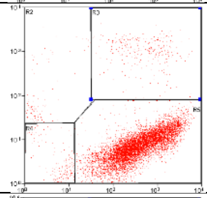	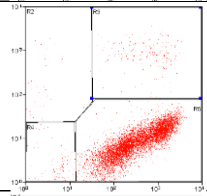	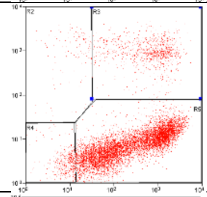
0.5 µg/mL	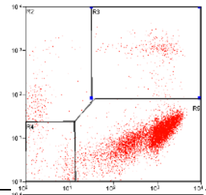	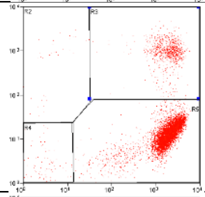	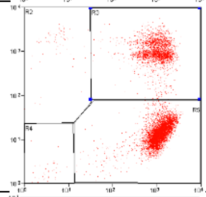	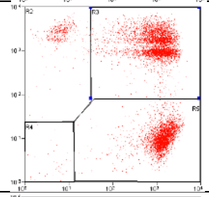
1 µg/mL	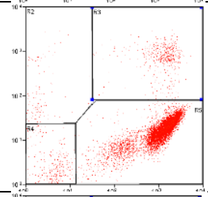	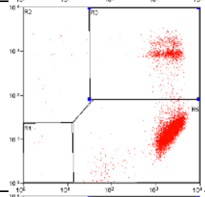	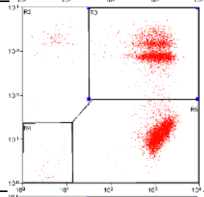	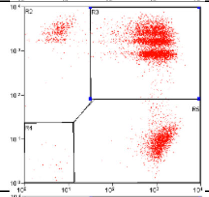
2 µg/mL	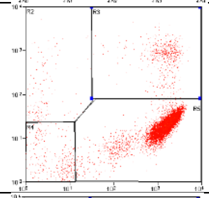	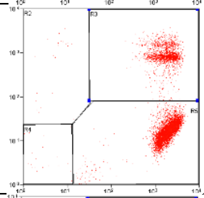	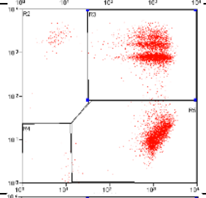	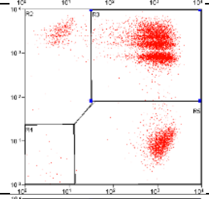
4 µg/mL	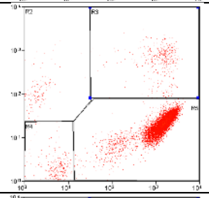	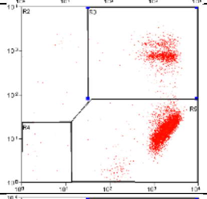	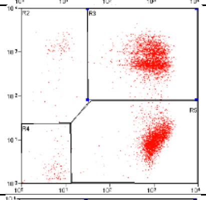	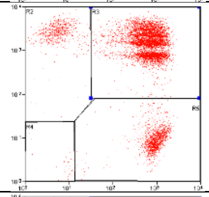
8 µg/mL	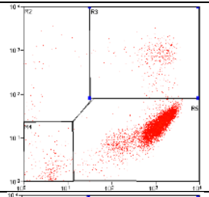	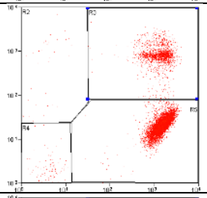	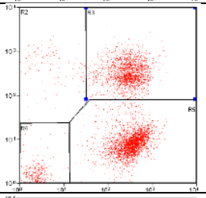	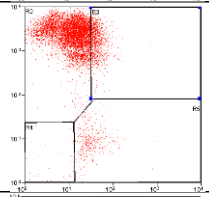
16 µg/mL	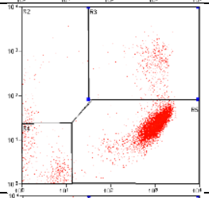	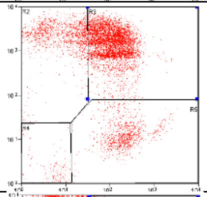	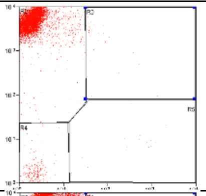	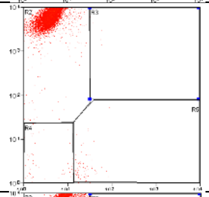
32 µg/mL	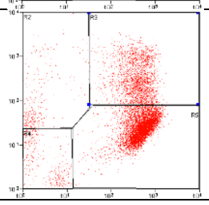	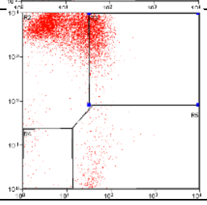	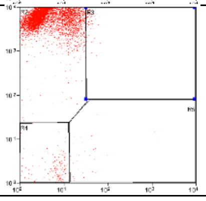	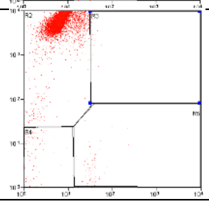

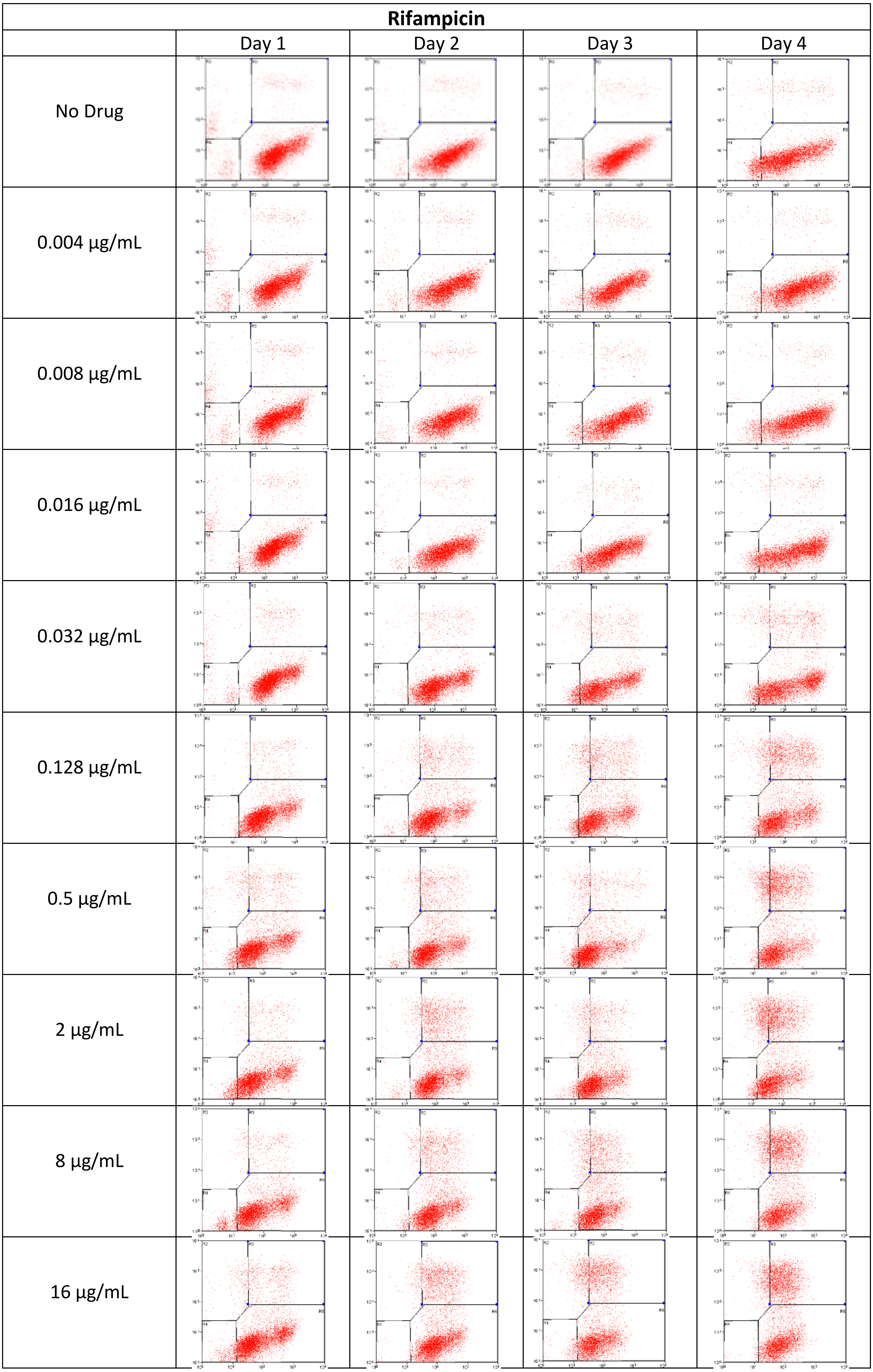

Figure S2 Representative dual parameter flow cytometry plots for *M. tuberculosis* cells that have been exposed to a range of concentrations of either isoniazid or rifampicin over a 4 day time-course
